# War Metaphors in Political Communication on Covid-19

**DOI:** 10.3389/fsoc.2020.583680

**Published:** 2021-01-25

**Authors:** Eunice Castro Seixas

**Affiliations:** Research Centre in Economic and Organizational Sociology (SOCIUS), Research in Social Sciences and Management (CSG), Lisbon School of Economics and Management (ISEG), University of Lisbon, Lisbon, Portugal

**Keywords:** war metaphors, crisis communication, political communication, COVID-19, critical discourse analysis

## Abstract

Although militaristic metaphors have been pervasive during health crisis in political and science communication, few works have examined how these linguistic devices may influence crisis communication. Drawing on critical discourse analysis (CDA) and on crisis communication literature, I show how political representatives have used the war metaphor for very different purposes in terms of crisis communication and management of the current Covid-19 pandemic. I suggest that these findings challenge previous criticisms of the war metaphor as inherently negative and damaging. Finally, I discuss possibilities of using CDA, and specifically, metaphor analysis to inform and expand crisis communication.

## Introduction

Militaristic metaphors have long been used in health crisis in political and media communicative practices, namely in relation to former epidemics, like the Severe Acute Respiratory Syndrome—SARS (Chiang and Duann, 2007, p. 587–597; Joye, 2010, p. 594; Koteyko et al., 2008, p. 247–259) and the Avian flu (de la Rosa, 2007, p. 18–26; de la Rosa, 2008, p. 91–94), but also regarding cancer and AIDS. This metaphorical use has been widely criticized, specifically: for being “inherently masculine, power-based, paternalistic and violent” (Reisfield and Wilson, 2004, p. 4025); contributing to the passivization of the patient; adding to further anxiety and stigmatization by blaming the victims when these are not able to win the battle, promising a victory that may be illusory and paving the way for the acceptance of the violence of the treatment and potential “collateral damages” (Hodgkin, 1985, p. 1820; Ross, 1989, p. 55; Stibbe, 1997, p. 68, 69; Sontag, 1979, p. 64–66; Sontag, 1989, p. 94).

In spite of the relevance of these criticisms, it is important to note that, as language is embedded in societal, political, and ideological structures and processes, the meaning of the signifiers depends on the specific relations between text and context. On the other hand, the recurrent use of militaristic metaphors during health crises may be actually a proof of its effectiveness as a rhetorical tool, which is something that in itself deserves further critical scrutiny. In this respect, although many discourse analysts have shown the importance of metaphors in political communication, few works have examined how these linguistic devices may actually aid or conversely, hinder, crisis communication, and management. Such an endeavor could be important in promoting a more critical approach to crisis communication, as well as in widening the application of critical discourse analysis (CDA) and specifically, metaphor analysis, to the understanding of crisis situations.

It is against this backdrop that I analyze the use of militaristic metaphors by political representatives during the current Covid-19 pandemic. From the beginning of the emergence of the Covid-19 virus in Europe we have seen the proliferation of militaristic metaphors of war and battle, with the current situation being frequently described as a “war,” health professionals being compared to front line soldiers, and the need for making sacrifices in these difficult and exceptional times being emphasized by several spokespersons. These and similar metaphorical use are contributing to “doing” different things, in terms of crisis communication and management, with implications in terms of biosecurity and biopolitics, but also in geopolitics. Indeed, the findings of this study show how, within the context of Covid-19, war metaphors were important in: preparing the population for hard times; showing compassion, concern and empathy; persuading the citizens to change their behavior, ensuring their acceptance of extraordinary rules, sacrifices; boosting national sentiments and resilience, and also in constructing enemies and shifting responsibility. By revealing the different uses of such militaristic metaphors, I also show how the context is itself construed and controlled differently by the political representatives.

I start by presenting some introductory reflections on the literature on crisis communication and its limitations to reflect subsequently on the challenges of communication and management of Covid-19 crisis. Next, I outline the methodological approach used in this paper drawing from both CDA and crisis communication studies. Then, I present the findings of the analysis, showing how militaristic metaphors are used by spokespersons for managing this health crisis in very specific and differentiated ways. Finally, I discuss the main findings and the implications of the prevalence of militaristic metaphors in crisis situations, and argue for the development of a line of research linking crisis communication with CDA and metaphor analysis.

## COVID-19 and Crisis Communication

Much of the literature on crisis communication emphasizes organizations' practices of reputational management for the effectiveness of their response to the crisis (Benoit, 1995, p. 13–62; Benoit, 1997, p. 182–185; Coombs, 2007, p. 37; Coombs and Holladay, 2009, p. 2–5; Kim and Sung, 2014, p. 62, 63; Lyon and Cameron, 2004, p. 217–219; Ma and Zhan, 2016, p. 102–105; Payne, 2006, p. 165, 166; Zheng et al., 2018, p. 58). Nevertheless, crisis communication has a variety of goals, which are not restricted to limiting reputation damage, some of the most important being: reducing harm, reestablishing public order and protecting the public. Some of these goals may at times conflict and the different actors involved, such as governmental agencies, corporations, the media, or the public, may prioritize different goals (Seeger, 2006, p. 234). Furthermore, although Situational Crisis Communication Theory (SCCT) considers crisis as reputation threats, Coombs (2007, p. 165) suggests a consideration of the ethical aspects in managing a crisis “To be ethical, crisis managers must begin their efforts by using communication to address the physical and psychological concerns of the victims. It is only after this foundation is established that crisis managers should turn their attentions to reputational assets.”

Taking a different research perspective, some authors (for example, Covello, 2003; Seeger, 2006, p. 232–234) have sought to study and describe best practices in crisis communication, that is practices thought to improve the effectiveness of crisis communication, especially within the context of large publicly-managed crises. These include: process approaches that combine both crisis and risk communication and whereby “communication strategies are fully integrated into the decision-making process” (Seeger, 2006, p. 236); pre-event planning; partnerships with the public; listening to the public's concerns and understanding the audience; honesty, candor and openness; collaboration and coordination with credible sources; meeting the needs of the media; communicating with compassion, concern and empathy; accepting uncertainty and ambiguity and providing self-efficacy messages (Seeger, 2006). This work is relevant to show how strategic communication during crisis situations goes beyond mere reputation management. Additionally, although most of the scholars working on crisis communication have been focusing on various types of organizations, an effective crisis communication and management is key in different types of crisis, including the Covid-19 pandemic, which can be considered as a large publicly-managed crisis.

In terms of crisis communication and management, Covid-19 pandemic has been quite challenging for several reasons, some of these related with previously mentioned factors affecting the effectiveness of communication in crisis situations, but also because of political, economic, and sociocultural factors that are not usually considered in crisis communication literature.

Firstly, although crisis are inherently dynamic and unpredictable (Seeger, 2006, p. 234), some, like this one, are more unpredictable than other. Indeed, although we know that this is a virus from the family of the coronavirus, we are in fact dealing with a knew and therefore unknown and unpredictable virus that presents itself through a wide array of symptoms, but remains asymptomatic and thus invisible in a percentage of the population. In spite of the enormous effort that is being made by the global scientific community to study Covid-19 in real time and come up as fast as possible with an effective treatment, the amount of uncertainty regarding this virus and its consequences is indeed tremendous. As there is no vaccine and therefore no official effective treatment for the virus it is also difficult to predict the end of the pandemic and fears of a second wave of the virus constraint the process of decision-making regarding the removal of the exceptional measures taken to contain the spreading of the disease and the reopening of the society and economy.

Secondly, the sudden outburst of cases of persons infected with Covid-19 that required hospitalization has highlighted the deficiencies of many national health systems and their unpreparedness and lack of capacity to deal with such a pandemic. This happened more harshly in some European countries, like in Italy and Spain, but also in UK whose health-systems had been suffered badly from austerity measures. But the fact that these countries were very late in their response to the crisis also contributed to this problem. Other European countries that were similarly hit by austerity measures and also had an aging population (like Portugal and Greece^1^) appeared to have learned from these experiences and attempted to delay the peak of the pandemic as they gained time to prepare their health systems, as well as the population to deal with the pandemic. An awareness that their national health systems wouldn't survive the peak of the pandemic was probably chief in their early response to the crisis. Nonetheless, the type of measures and the timing of these responses have varied greatly from country to country and their adequacy is yet to be determined.

Thirdly, this health crisis highlighted the importance of taking into consideration sociocultural factors impacting specifically, in this case, on the level of compliance of the population regarding social distancing, a key measure to avoid the spreading of the virus. Indeed, in Europe, for example, southern and Mediterranean cultures had to make an extra effort to change their habits and automatic gestures regarding greetings and body contacts compared to Northern countries. Besides these differences in customs and habits, citizens tendency to trust and comply or, conversely, to be suspicious of and resist governmental demands may also vary greatly from country to country depending on historical, political and cultural factors. The cultural importance of health, particularly in the Greek culture, was also considered a motivation for the acceptance of lock-down measures, as health was prioritized over the economy (Perrigo and Hincks, 2020). Unfortunately, these factors have seldom been taken into consideration in crisis communication literature.

Fourthly, Covid-19, like other crisis, has brought to the surface the fragility of many societies and economies and particularly, the social inequalities that make some social groups more vulnerable to the health crisis and/or to the subsequent economic crisis. This has been more salient regarding access to health care, housing conditions, and the impact of lock-down on the poorer and the workers of gray economy.

Fifth, governmental agencies in charge of managing the crisis had to deal with an intensified media scrutiny and also with the proliferation of fake news in social media. Acknowledging the key role of the media in crisis communication required thus an emphasis on remaining accessible and fostering a dialogic communication with the public, accepting it as a legitimate and equal partner as Seeger (2006, p. 238) suggests. Moreover, it became crucial also to deconstruct the growing disinformation on Covid-19 that emerged in the social media in order to protect the public.

And finally, this pandemic has also highlighted the lack of coordination at the global and regional levels to deal with the pandemic. Instead of solidarity and the development of a common strategy for “fighting” the virus, we have too often witnessed the rise of geopolitics and competition for health resources between the powers. At the EU level the lack of a timely response due to disagreement between the powers has been once again evident. So much that many European countries had to count on China and also Russia, for donations of protection material, ventilators, as well as, in most severe cases, like in Italy, teams of health care professionals and experts in pandemics.

As all crisis can be thought of as opportunities, Covid-19 pandemic can also be considered an opportunity to rethink and expand crisis communication theories. In the following section, I suggest doing so by using critical discourse analysis and specifically critical metaphor analysis, to inform and expand crisis communication studies.

## Methodology: Using CDA With Crisis Communication Studies

“Discourse analysis can shed light on the texts that lead to, surface during, collide and become refined after a crisis” (Heath, 2010 p. 3).

CDA is a qualitative orientation to discourse analysis associated to the study of power dynamics in society and its main goal is to analyze how power is enacted, reproduced and resisted, through text or speech (Fairclough, 2001, p. 43). Methodologically, this paper is inspired by recent work suggesting that CDA can be used to inform crisis communication. Specifically, CDA has been used to promote a critical political-economic evaluation of the communicative practices during crisis situations (Alexander, 2013, p. 1), and to giving voice to silenced/alternative narratives (Dunn, 2010, p. 1–4; Dunn and Eble, 2015, p. 732, 733). Other relevant work on the “rhetorical arena” has sought to advance a multi-vocal approach to crisis communication, which postulates that crisis publics (receivers) can also become crisis communicators (Frandsen and Johansen, 2010, p. 428; Coombs and Holladay, 2014 p. 41).

The main objective of the analysis presented here is to explore how political representatives use militaristic metaphors during the present Covid-19 epidemic, in order to manage the crisis. As my focus is on the goals and strategic dimensions of communicative practices of spokespersons in the context of the Covid-19 pandemic, I draw heavily from CDA interest in the strategic dimension of discourse. The latter understands language as “a goal oriented activity taking place amid a set of contextual constraints” (Ihnen and Richardson, 2011, p. 235). Building from such perspective, less attention will be given in this analysis to aspects of textual coherence and cohesion and more attention will be paid to the social conditions of the production and interpretation of the text, namely: the intertextual and interdiscursive relationships between utterances, texts and discourses; the specific context of the situation in social and institutional terms and the broader socio-political and historical context. The latter aspects correspond respectively, to the second, third and fourth levels of context as proposed by the DHA of CDA (Reisigl and Wodak, 2001, p. 93).

I draw also from various work within CDA that has focused on the use of metaphors in political and media discourse. Metaphors are crucial for expressing attitudes and beliefs and making sense of complex events (Lakoff and Johnson, 1980, p. 156–160). They are especially important in political discourse as interpersonal devices that facilitate the creation of a relationship with the public, being often used with a persuasive function (Charteris-Black, 2004, p. 7–13; Charteris-Black, 2009, p. 103; Ferrari, 2007, p. 621; Kitis and Milapides, 1997, p. 562, 563). Metaphors are thus key linguistic devices for constructing social relations and creating, contesting or legitimating specific social, cultural or political and ideological representations of the world (Charteris-Black, 2004, p. 8; Fairclough, 2001, p. 120; Musolff, 2012, p. 303, 304; Zinken, 2003, p. 519, 520).

Metaphors are also crucial cultural and linguistic tools for conceptualizing disease. Some of this work has focused on Severe Acute Respiratory Syndrome (SARS) revealing the persistence of sub-war metaphors (Larson et al., 2005, p. 263), and the importance of the social and political context for SARS metaphorical framing (Wallis and Nerlich, 2005, p. 2638; Chiang and Duann, 2007, p. 589–595). Other relevant work on Avian flu has highlighted the use of metaphors as rhetorical and persuasive devices (de la Rosa, 2008, p. 28, 29), and shown how the war metaphor could be used for portraying a global fight against this disease (de la Rosa, 2007, p. 16, 17).

In this paper, and in line with a focus on a strategic dimension of discourse, I approach metaphors “as actions that are embedded in larger discursive activities” (Zinken and Musolff, 2009, p. 2), and also as “matters of speaker choice” (Charteris-Black, 2004, p. 10). For this analysis, I selected a sample of speeches given by key political representatives during the Covid-19 epidemic, focusing on the month of March 2020, when most of the countries have put forward more restrictive measures to deal with pandemic. These speeches were selected on the basis of their (predominant) use of militaristic metaphors, and include speeches of Marcelo Rebelo de Sousa, the President of Portugal, Emmanuel Macron, the President of France, Boris Johnson, the Prime-Minister of UK, Pedro Sánchez, the Prime-Minister of Spain, Ursula von der Leyen, the President of the European Commission, Donald Trump, the President of the US and António Guterres, the Secretary-General of the United Nations. The full speeches were accessed from official websites in its original language. They were read repeatedly and coded according to their use of militaristic metaphors and/in relation to crisis communication and management. The analysis evidenced seven different ways of managing the crisis through the use of militaristic metaphors, which are presented in the next section. Extracts (presented here in the original language and its translation to the English language) were selected for their relevance to evidence these different associations with crisis communication and management.

## Findings: Managing the Crisis Through Militaristic Metaphors

The analysis reveals that the war metaphor not only appears in the analyzed speeches, it also tends to be the main organizing theme of the text, forming the backbone of its argumentative and rhetorical strategies. Moreover, this metaphor aids in the managing of the health crisis through the pursuit of specific goals such as: preparing the public for hard times; persuading citizens to change their behavior; fostering national unity, mobilization and resilience; showing compassion, concern and empathy; avoiding responsibility and mitigating blame and constructing enemies and shifting blame and responsibility. Additionally, the example of speeches made by António Guterres, the Secretary-General of the UN is presented here because it shows how the war metaphor can also be used to promote peace and justice.

### Preparing the Public for Hard Times

Most of the political representatives that used militaristic metaphor to talk about Covid-19 were doing so as part of preparing the public for hard times, by asserting the seriousness of the situation and also in order to legitimate exceptional measures such as the declaration of state of alarm, state of emergency, or lock-down of the country or some of its regions. The war metaphor facilitates the public understanding that the situation is grave and hence public acceptance of exceptional measures and sacrifices. Moreover, as measures like the declaration of the state of emergency are usually linked to wartime memories, the use of militaristic metaphors gains further coherence within such statements. Often, the war metaphor appears at the beginning of the speech as a way of framing the situation and also repeatedly throughout this. Below I present some examples of this type of use of the war metaphor.

Marcelo Rebelo de Sousa, the President of Portugal, declares the state of emergency at 18 March 2020 and starts its speech by describing the situation as a war:

*Esta guerra – porque de uma verdadeira guerra se trata – dura há um mês, começou depois dos vizinhos europeus, e, também por isso, pôde demorar mais tempo a atingir os picos da sua expressão*.

*/This war - because it is a real war - has been going on for a month, it started after European neighbors, and for this reason, it could take longer to reach the peak of its expression*.

He continues to use militaristic terms such as “combat” as in the following statement:

*E os portugueses, com a experiência de quem já viveu tudo numa história de quase nove séculos, disciplinaram-se, entenderam que o combate era muito duro e muito longo e foram e têm sido exemplares*.

*/And the Portuguese, with the experience of those who have lived everything in a history of almost nine centuries, disciplined themselves, understood that the combat was very hard and very long and were and have been exemplary*.

Subsequently in this speech, the state of emergency is even re-signified to be depicted as a sign of democracy:

É* também um sinal democrático*.

*Democrático, pela convergência dos vários poderes do Estado*.

*Democrático, porque é a democracia a usar os meios excepcionais que ela própria prevê para tempos de gravidade excepcional*.

*Não é uma interrupção da democracia. É a democracia a tentar impedir uma interrupção irreparável na vida das pessoas*.

*/It is also a democratic signal*.

*Democratic, by the convergence of the various powers of the state*.

*Democratic, because it is democracy using the exceptional means that it itself envisages for times of exceptional gravity*.

*It is not an interruption of democracy. It is democracy trying to prevent an irreparable interruption in people's lives*.

Thus, by using militaristic metaphors, the President of Portugal is effectively preparing the Portuguese for exceptional times (“a real war”; a “very hard and very long combat”) and hence, for the acceptance of exceptional measures such as the state of emergency. At the same time, Marcelo Rebelo de Sousa evokes the long history of Portugal to praise the resilience and expected compliance of the Portuguese with these exceptional measures. And finally, the speaker even re-signifies the meaning of the state of emergency in order to delink it from any idea of chaos or totalitarianism and link it with democracy itself. This discourse was praised by the newspaper “Observador” as “the best speech of Marcelo's life” and in general, the Portuguese citizens did show compliance for the state of emergency measures.

The French President, Emmanuel Macron, has uttered the word “war” seven times in his televised speech on the 16th of March, 2020. His use of the war metaphor also precedes the announcement of specific measures to fight the pandemic such as: the suspension of all undergoing reforms; “a new bill allowing the government to respond to emergencies and, where necessary, to legislate by ordinance,” and the decision taken to close the border with the EU and the Schengen area.

*Nous sommes en guerre. Toute l'action du gouvernement et du Parlement doit être désormais tournée vers le combat contre l'épidémie, de jour comme de nuit. Rien ne doit nous en divertir. C'est pourquoi j'ai décidé que toutes les réformes en cours seraient suspendues, à commencer par la réforme des retraites*.

*Dès mercredi, en conseil des ministres, sera présenté un projet de loi permettant au gouvernement de répondre à l'urgence et, lorsque nécessaire, de légiférer par ordonnance dans les domaines relevant strictement de la gestion de crise. Ce projet sera soumis au Parlement dès jeudi. J'ai vu tout à l'heure les présidents de l'Assemblée nationale et du Sénat afin que ces textes soient votés le plus finement possible, afin aussi que la vie démocratique et le contrôle du Parlement continuent dans cette période. Je les en remercie et je remercie tous nos parlementaires en cet instant*.

...

*Nous sommes en guerre. Aussi, comme je vous l'ai dit jeudi, pour nous protéger et contenir la dissémination du virus, mais aussi préserver nos systèmes de soins, nous avons pris ce matin, entre Européens, une décision commune. Dès demain midi, les frontières à l'entrée de l'Union européenne et de l'espace Schengen seront fermées. Concrètement, tous les voyages entre les pays non européens et l'Union européenne seront suspendus pendant trente jours*.

*/We are at war. All the action of the government and of Parliament must now be turned toward the fight against the epidemic, day and night. Nothing can divert us. That is why I decided that all the ongoing reforms would be suspended, starting with the pension reform*.

*On Wednesday, in council of ministers, a bill will be introduced allowing the government to respond to emergencies and, where necessary, to legislate by ordinance in areas strictly related to crisis management. The draft will be submitted to Parliament on Thursday. I saw the Speakers of the National Assembly and the Senate earlier so that these texts could be voted on as finely as possible, so that democratic life and the control of Parliament would continue in this period. I thank them for that, and I thank all our parliamentarians at this time*.

...

*We are at war. So, as I told you on Thursday, to protect us and to contain the spread of the virus, but also to preserve our health care systems, we made a joint decision this morning among Europeans. From tomorrow noon, the borders at the entrance of the European Union and the Schengen area will be closed. In concrete terms, all travel between non-European countries and the European Union will be suspended for 30 days*.

The repeated statement “We are at war” frames the announcement of these decisions, contributing to legitimating the exceptional measures taken. Such framing is reinforced in the first paragraph by the statement: “All the action of the government and of Parliament must now be turned toward the fight against the epidemic, day and night. Nothing can divert us.”

### Persuading Citizens to Change Their Behavior

Linked with the previous goal, the use of militaristic metaphors by political representatives not only serves for preparing the public to accept exceptional measures, but it is also very important to persuade citizens to change their behavior in accordance to these measures. Ensuring compliance is promoted discursively through the combine use of what can be characterized as soft and hard power devices, as when spokespersons ask the public to behave in a certain way (*soft power*), and at the same time, declare that there will be legal consequences if citizens fail to comply (*hard power*).

For an example of such a metaphorical use, one can look at the way that Boris Johnson, the UK's Prime-Minister, presents the virus as an “invisible killer” and explains what British people can to help in fighting the disease. The speaker gives the British people “an instruction” to stay at home. His use of deontic modalities (“I must give;” “we must do;” “people will only be allowed”) assists in defining what is necessary or possible under this fight and to the speaker's goal of persuading citizens to change their behavior.

*Good Evening*,

*The coronavirus is the biggest threat this country has faced for decades – and this country is not alone*.

*All over the world we are seeing the devastating impact of this invisible killer*.

*And so tonight I want to update you on the latest steps we are taking to fight the disease and what you can do to help*.

...

*From this evening I must give the British people a very simple instruction - you must stay at home*.

*Because the critical thing we must do is stop the disease spreading between households*.

*That is why people will only be allowed to leave their home for the following very limited purposes*.

### Fostering National Unity, Mobilization, and Resilience

In crisis communication, it is important to insure the unity and resilience of the community in order to maintain morale and mobilize people to assist in the management of the crisis. The metaphor of war also helps promote a “general mobilization” of the citizens in order to fight the progress of this “invisible enemy.” In the Covid-19 pandemic, several political leaders have used this metaphor to appeal to unity and call for a mobilization of the population.

Emmanuel Macron has characterized the virus as an invisible and elusive enemy, presenting it in this way as a hefty threat that requires “our general mobilization.” The use of the possessive “Notre/Our,” as well as the repeated use of the pronoun “Nous/We” strengthens this appeal for unity among the French citizens against this common enemy.

*Nous sommes en guerre, en guerre sanitaire certes. Nous ne luttons ni contre une armée ni contre une autre nation, mais l'ennemi est là, invisible, insaisissable, et qui progresse. Et cela requiert notre mobilization générale*.

*We are at war, certainly in a health war. We are not fighting against one army or another nation, but the enemy is there, invisible, elusive, and progressing. And that requires our general mobilization*.

It is important, however, to analyze these statements in terms of what is silenced by these (the relation of the text with the context). In this regard, Macron's suggestion of a shared identity with the audience (the French citizens) and of a national sense of unity stands in sharp contrast with the magnitude of the protests of Yellow Vests (Gillets Jaunes) that emerged in 2018, triggered by fuel tax rises, and expanded into a revolt against Macron's government.

Marcelo Rebelo de Sousa, the President of Portugal, also mentions an invisible and insidious enemy, but he is not referring to the virus. Instead, he characterizes this enemy as one that is present in every war and that has several names: “discouragement, tiredness and time fatigue that never ends.” This is a call for resilience in times of “war,” a call for “resistance, solidarity and courage” that resembles another speech, the one of Franklin D. Roosevelt, the 32nd President of the U.S. in his first inaugural address, at 4 March, 1933. In this speech, Roosevelt uttered a statement that would become famous: “The only thing we have to fear is fear itself.”

*Termino com um pedido*.

*Nesta guerra, como em todas as guerras, só há um efectivo inimigo, invis*í*vel, insidioso e, por isso, perigoso*.

*Que tem vários nomes*.

*Desânimo. Cansaço. Fadiga do tempo que nunca mais chega ao fim*.

*Temos de lutar, todos os dias, contra ele*.

*Contra o desânimo pelo que corre mal ou menos bem*.

*Contra o cansaço de as batalhas serem ainda muitas e parecerem dif*í*ceis de ganhar*.

*Contra a fadiga que tolhe a vontade, aumenta as dúvidas, alimenta indignações e revoltas*.

*Tudo o que nos enfraquecer nesta guerra alongará a luta e torná-la-á mais custosa e dolorosa*.

*Resistência, solidariedade e coragem são as palavras de ordem*.

/*I'll end with a request*.

*In this war, as in all wars, there is only one real enemy, invisible, insidious and therefore dangerous*.

*It has many names*.

*Discouragement. Tiredness. Fatigue of time that never comes to an end*.

*We have to fight him every day*.

*Against discouragement for what goes wrong or less well*.

*Against the fatigue of the battles still being many and seem hard to win*.

*Against the fatigue that kills the will, increases doubts, feeds indignations and revolts*.

*Whatever weakens us in this war will lengthen the fight and make it more costly and painful*.

*Resistance, solidarity and courage are the watchwords*.

Ursula von der Leyen, the president of the European Commission, also uses the war metaphor to send a message of self-efficacy to the EU, by explaining what “every single one of us” can do to fight the virus and call for a general mobilization in this fight. Interesting in this speech is the use of an economic metaphor of “debt” that here is used in moral terms to express the debt of gratitude toward the health professionals.

*But what is unique about this fight is that every single one of us has a role to play. Every single one of us can help repay that debt. By keeping our distance we can slow down the spread of the virus. The numbers in the last few days have shown that we can bend the trend – but only if we all do our share*.

### Showing Compassion, Concern, and Empathy

In crisis situations, communicating with “appropriate levels” of compassion, concern and empathy is known to increase the credibility of the message and enhancing the perceived legitimacy of the messenger (Coombs, 2007, p. 172; Seeger, 2006, p. 241). Spokespersons have often shown compassion for the victims of the Covid-19 and their families, and acknowledgment of the resilience required to endure lock-down measures.

Ursula von der Leyen, for example, has expressed compassion for the victims of the pandemic “currently fighting for their lives,” as well as their “loved ones.” In this speech, the metaphor of “fight” is used to express the harshness of a patient's individual struggle with the disease, and directed at the persons infected by the virus.

*My heart goes out to all of the victims and their loved ones*.

*And all of our thoughts and best wishes are with those currently fighting for their lives or sick at home*.

Emmanuel Macron has also expressed empathy, but in this case, regarding the impact of the exceptional measures on the lives of the French citizens, and the difficulty of changing one's habits.

*Mes chers compatriotes, je mesure l'impact de toutes ces décisions sur vos vies. Renoncer à voir ses proches, c'est un déchirement. Stopper ses activités quotidiennes, ses habitudes, c'est très difficile. Cela ne doit pas nous empêcher de garder le lien, d'appeler nos proches, de donner des nouvelles, d'organiser aussi les choses avec nos voisins. D'inventer de nouvelles solidarités entre générations. De rester, comme je vous l'ai dit jeudi dernier, profondément solidaires et d'innover là aussi sur ce point. Je sais que je vous demande de rester chez vous*.

*My fellow countrymen, I see the impact of all these decisions on your lives. Giving up seeing your loved ones is heartbreaking. It's very difficult to stop your daily activities and habits. This should not prevent us from keeping the link, calling our relatives, giving news, also organizing things with our neighbors. From inventing new solidarity between generations. From remaining, as I told you last Thursday, deeply in solidarity and to innovate there too on this point. I know I'm asking you to stay home*.

By first recognizing the sacrifices requested from the French citizens, and putting himself in this same group—by using the pronoun “nous”/we—the speaker can then present the situation as an opportunity: for “inventing” and “innovating” in issues of solidarity.

The idea of exceptional measures and the need to make sacrifices evokes wartime. Although this specific extract does not include any militaristic metaphor, it has to be understood in the context of the entire speech. All Macron's speech is organized around this idea of being in a war, the pandemic being compared to a war situation (see section Preparing the Public for Hard times). And this is foregrounded in the beginning of the speech:

*Jamais la France n'avait dû prendre de telles décisions, évidemment exceptionnelles, évidemment temporaires en temps de paix*.

*/France has never had to make such decisions, obviously exceptional, obviously temporary in peacetime*.

Thus, the need to make sacrifices, just like in wartime, had already been put forward by Macron earlier in his speech, allowing at this moment for the speaker to show empathy regarding the impact of the exceptional measures on the lives of citizens.

### Avoiding Responsibility and Mitigating Blame

In a long speech of 195 min, which was widely criticized by the Spanish media, Pedro Sanchez, the prime-minister of Spain, presents the virus as an unknown enemy, “un enemigo al que aún estamos conociendo”/“an enemy we are still getting acquainted with” (Pedro Sanchez, 21 March 2021). Besides “enemigo” (enemy), Sanchez uses several other words conveying a militaristic metaphor such as: “resistencia” (resistance), “lucha” (fight), and “batalla” (battle).

In terms of crisis communication, the goal of such message is clearly that of avoiding responsibility and mitigating blame for the lack of control of the pandemic. While it is true that Covid-19 is in fact a new virus and therefore unknown, the focus on the idea of lack of control masks the failure of the Spanish government to provide a timely and effective response to contain the damage and protect the population. In terms of crisis communication, failure in acknowledging the government's responsibility corresponds to a lack of candor on the part of the speaker, understanding this as “communicating the entire truth as it is known, even when the truth may reflect negatively on the agency or organization” (Seeger, 2006, p. 239). Indeed, although to acknowledge the uncertainty of the situation can be considered a best practice in crisis communication (Seeger, 2006, p. 241, 242), that cannot serve as an excuse for not communicating the entire truth.

Increasingly weakened politically, Pedro Sanchez will later apologize (on 20 May) for his mistakes in managing the pandemic, although he further justifies these by the “urgency of times, scarcity of resources and exceptional nature and absence of precedents.”

### Constructing Enemies and Shifting Blame and Responsibility

Following weeks of downplaying the seriousness of the pandemic, and as the virus spread in the country, Donald Trump, the President of the US, also engaged in wartime rhetoric and even called himself “a wartime President:” “I view it as a, in a sense, a wartime President. I mean, that's what we're fighting” (18 March 2020). In this statement, Trump emphasizes the sacrifices necessary in such times, such as the one of closing part of the economy: “One that you have to close it down in order to defeat this enemy. But we are doing it. And we are doing it well.”

Such militaristic metaphors were followed on the following day (19 March 2020) by a naming of the Covid-19 as the “Chinese virus” as Trump made his speech in the Virus Task Force Hold Briefing: “We continue our relentless effort to defeat the Chinese virus.” Such an association of diseases with a foreign place and other has a long tradition and “reveals a link between imagining disease and imagining foreignness” (Sontag, 1989, p. 47–55).

What this rhetoric suggests, is an attempt by Donald Trump to shift blame and responsibility for the pandemic by focusing the attention on China. Moreover, Trump has also blamed the WHO for allegedly aligning uncritically with China's narrative regarding the origin of the Covid-19.

These attempts to shift responsibility can be understood as scapegoating, a primary crisis response strategy predicted by SCCT, through which “the crisis manager blames some person or group outside of the organization for the crisis” (Coombs, 2007, p. 170). Shifting the focus of the attention and controlling the discourse is also a way of avoiding the journalists' distressing questions regarding the seriousness of the situation in the US, which the president has repeatedly sought to deny. In addition to denial—of both of the seriousness of the crisis and his responsibility in failing to provide an adequate response to it, Trump has recurrently used another reputation management strategy: the one of emphasizing his current good deeds, what Kim and Liu (2012, p. 82) have called “enhancing.” This discursive device differs from “bolstering”—emphasizing the corporation past good deeds that has been suggested by SCCT (Coombs, 2007, p. 172).

Thus, in his communicative practices regarding Covid-19 Donald Trump has predominantly used reputation management strategies rather than strategies focused on helping the public deal physically and psychologically with the pandemic. The focus on reputation management evokes crisis communication strategies adopted by US corporations during the 2009 flu pandemic, which were in contrast to government organizations' response that emphasized providing instructing information to the public, such as guidelines about how to respond to the crisis (Kim and Liu, 2012).

### A Call for Peace

Finally, on a positive note, António Guterres, the ninth Secretary-General of the United Nations has also used the metaphor of war, referring to the virus as “a common enemy,” which in this context seems to imply a global enemy, that attacks all people in the world. However, somewhat paradoxically, the war metaphor is used here by the speaker to call for a global ceasefire:

*Our world faces a common enemy: COVID-19*.

*The virus does not care about ethnicity or nationality, faction or faith. It attacks all, relentlessly*.

*Meanwhile, armed conflict rages on around the world*.

*The most vulnerable — women and children, people with disabilities, the marginalized and the displaced — pay the highest price*.

*They are also at the highest risk of suffering devastating losses from COVID-19*.

*Let's not forget that in war-ravaged countries, health systems have collapsed*.

*Health professionals, already few in number, have often been targeted*.

*Refugees and others displaced by violent conflict are doubly vulnerable*.

*The fury of the virus illustrates the folly of war*.

*End the sickness of war and fight the disease that is ravaging our world*.

*That is why today, I am calling for an immediate global ceasefire in all corners of the world*.

*It is time to put armed conflict on lockdown and focus together on the true fight of our lives*.

*(*António Guterres, 23 March 2020).

In this speech, Guterres argues that the war has made people, health-systems and countries more vulnerable, and hence more defenseless also to the attack of the virus. Thus, he effectively shifts the attention away from the virus and its victims to the war and its systemic impacts in creating vulnerable groups (women and children, people with disabilities, the marginalized and the displaced) that are also the ones at the highest risk of Covid-19. The speaker goes on framing the audience perspective on both war and the virus. This is made by comparing “the fury of the virus” with “the folly of war” and using the metaphor of disease to describe the war “End the sickness of war and fight the disease that is ravaging our world.” Finally, Guterres uses the lock-down as a metaphor for closing down the war.

Subsequently, on the 26 March 2020, the Secretary-General of the United Nations warns that we are not winning this war against Covid-19 and presents the numbers to support his argument.

*We are at war with a virus – and not winning it*.

*It took the world 3 months to reach 100,000 confirmed cases of infection*.

*The next 100,000 happened in just 12 days*.

*The third took 4 days*.

*The fourth, just one and a half*.

*This is exponential growth and only the tip of the iceberg*.

*This war needs a war-time plan to fight it*.

*Solidarity is essential. Among the G-20 – and with the developing world, including countries in conflict*.

*That is why I appealed for a global ceasefire*.

*(*António Guterres, 26 March 2020).

The metaphor of the war against the virus is invoked here to suggest that the solution must also be a solution tailored to the situation, that is, a “war-time plan,” which Guterres sees as founded on solidarity among the most powerful and peace.

## Concluding Remarks: Militaristic Metaphors, Political Communication, and CRISIS Management

The use of militaristic metaphors in health crisis is not new and with the emergence of the Covid-19 pandemic we have again witnessed its recurrent use by political representatives and by the media, particularly in television. In this paper I sought to explore the use of the war metaphor by political actors in its intersection with the practices of crisis communication and management. Drawing from the approach of CDA, and particularly the studies on the use of metaphors in political discourse and crisis communication, I suggest that such an approach can serve both to inform crisis communication literature and to develop CDA. This is in line with Chiapello and Fairclough's understanding of a transdisciplinary approach, which asks “how a dialogue between two disciplines or frameworks may lead to a development of both through a process of each internally appropriating the logic of the other as a resource for its own development” (Chiapello and Fairclough, 2002 cited in Fairclough, 2005, p. 53).

The findings show that first, the war metaphor was used often in the context of the recent pandemic of Covid-19, but also that it was used in very different ways in terms of crisis communication and management. Some political representatives have at times, used the war metaphor for purposes such as showing compassion, concern and empathy with the public and promoting self-efficacy and resilience in coping with the pandemic, which can be linked with recognized best practices in crisis communication. And the war metaphor is used by the Secretary-General of the United Nations, António Guterres to paradoxically, call for a global ceasefire and highlight the systemic impacts of war. Nonetheless, as my goal was just to explore and show the different uses of the war metaphor, rather than analyzing in detail the political communication and crisis management strategies of these representatives, from these good examples, it is not possible to conclude that these representatives were always ethical and followed best practices in the way they communicated and managed the Covid-19 crisis.

Furthermore, the findings of this study also suggest that the war metaphor is often used for the pursuit of specific goals of crisis communication and management such as: preparing the public for hard times, persuading the population to change their behavior and bolstering resilience and self-efficacy. These are messages that, while using the war metaphor, place the emphasis on adaptation to hard times, rather than on fighting an “invisible enemy.” Subsequent studies should try to go deeper in order to enable a comprehensive and critical analysis of the communication and crisis management strategies of each country or organization.

In general, these findings seem to caution against previous generalized criticisms of the war metaphor as inherently dangerous and damaging. Instead, they highlight the role played by the dialectics between text and context in discourse. Political discourse, which is the focus of this paper, is targeted to social groups. Hence, political use of the war metaphor raises the questions of what kind of categorizations are used by the spokespersons, who is included in this fight against the virus, and whose voices or alternative narratives are silenced. Constructions of self and other are also linked to differing ideological uses of the DISEASE IS WAR metaphor, as Chiang and Duann (2007, p. 581) have shown regarding SARS.

Addressing the issue of the strategic use of the war metaphor requires an understanding of the pragmatics of discourse, or, to put it simply, of what that speaker is doing in terms of political and crisis communication, while using the war metaphor. Is he/she, for example, trying to help citizens to cope physically and psychologically with the pandemics or is he/she attempting to engage in reputation management actions like avoiding or shifting responsibility for failures in the crisis management? Are some social groups being systematically neglected in terms of crisis management? These discursive actions only make sense if one understands the historical, social and political context of their occurrence. This means we need to analyze, as Fairclough (2001, p. 8) has argued, not only what people are doing with language and how language is linked to power, but also how and why they are doing it: “why are the facts as they are?; how - in terms of development of social relationships of power—was the existing sociolinguistic order brought into being?; how is it sustained?; and how it might be changed to the advantage of those who are dominated by it?”

In this case, it means also to situate the discursive act in the context of the global health crisis in order to analyze the way it has impacted and unfolded in that specific social context, nation or organization. In this regard, although Covid-19 was considered a pandemic, there has been a flagrant lack of coordination between nations even within the European Union. Further studies are needed to understand why this happened, why each nation followed its own strategy of crisis management and communication, but many have converged nonetheless on some measures of lock-down, social-distancing and protective use of masks.

It is useful here to look at the exceptions, at the cases where different crisis communication and crisis management strategies were used. Regarding the use of militaristic metaphors during the Covid-19 pandemic, it is worth noting Germany's discursive use of negation—denying that the Covid pandemic was a war. Following Macron speech of 16 March and against it, the president of Germany, Frank-Walter Steinmeier, declared: “It is not a war, it's a test to our humanity!” Such resistance in using militaristic metaphors may be linked to Germany's history, its participation in the WWII and responsibility for the inhumanity of holocaust. However, Germany's strategy for crisis management did not differ much from France's, in spite of this difference in framing the crisis. Sweden, on the other hand, in stark contrast with other European nations, has managed the epidemic by appealing to citizens' individual responsibility and accountability and relying on their trust in the state and expertise knowledge (Nygren and Olofsson, 2020, p. 3). In his address to the nation on 22 March, 2020, the Swedish Prime-Minister, Stefan Löfven, never once used the militaristic metaphor to refer to Covid-19. He was nonetheless engaging in crisis communicative practices with the goals of preparing the public for hard times, appealing to individual responsibility, bolstering resilience and self-efficacy and showing compassion and empathy with the public. These differences point to cultural and historical differences in the use of political language and specifically, militaristic metaphors.

Further studies should focus on the reception of these discourses in order to examine the effectiveness of the war metaphor in the various discursive actions associated with crisis management. Although some authors have sought to challenge crisis communication strong sender orientation (Frandsen and Johansen, 2010, p. 428; Coombs and Holladay, 2014, p. 41), more studies on this line are needed. Additionally, it can be important to examine situations when the use of militaristic metaphors may “backfire,” for example, when it becomes clear that the war against the virus is not being won, or that “collateral damages” of this war (be it the number of deaths or the number of unemployed and poor) becomes too great to be accepted by the public.

Finally, although this paper focuses on militaristic metaphors, these are often used by political representatives in conjunction with other discursive and rhetorical devices, inclusive other types of metaphors. Further studies should analyze how, in the context of Covid-19, other metaphors are being used alternatively to or in conjunction to the war metaphor, for example, the virus as a Killer metaphor, economic metaphors, patriarchal or religious metaphors. Such alternative metaphors, like the killer metaphor may be as problematic in their framing of the issue as militaristic ones (Wallis and Nerlich, 2005, p. 2634).

An interesting case is how Jair Messias Bolsonaro, the president of Brazil, has consistently denied the seriousness of the pandemic and positioned himself against any lock-down, confinement or social distancing measures by using androcentric and religious metaphors. In this regard, Bolsonaro has suggested that one should “face the virus like a man rather than a kid” in order to repudiate lock-down and social distancing measures. By doing so, he was criticizing anyone who attempted to protect themselves against the pandemic, blaming them for lack of courage and for behaving irresponsibly like “kids.” In fact, Bolsonaro's discourse has tended to equate responsibility with going to work as usual and irresponsibility with complying with lock-down, social distancing, and other protecting measures. Furthermore, when confronted by journalists with the rising numbers of the infected and dead in Brazil, he just replied, making a joke about his own name: “I am Messias but I do not perform miracles.” With such discursive act, the speaker was successfully evading the issue and at the same time, trivializing the impact of Covid-19, which is something he has also done in other occasions. The religious aspect is not a joke though as Bolsonaro has publicly appeared supporting the evangelical church in religious celebrations.

In this case, the populist facet of Bolsonaro's discourse drew heavily on religious, patriarchal and masculinity discourses. However, Bolsonaro also used the war metaphor in the context of Covid-19, but not for promoting measures of social distancing and protection against the virus. Instead, the war metaphor was used as a justification for the use of hydroxychloroquine and chloroquine, a highly controversial treatment. This discursive combination of militaristic and patriarchal metaphors is not new, but it is one that seems to be particularly invoked by populist leaders (Steinert, 2003, p. 267). However, unlike Steinert, who reinforces the view of the war metaphor as inherently negative and dominant, incorporating in itself elements of patriarchy and masculinity, the findings of these paper suggest taking a more critical look at the rhetorical and pragmatic elements of discourse and metaphorical use in political communication.

## Data Availability Statement

The original contributions presented in the study are included in the article/Supplementary Materials, further inquiries can be directed to the corresponding author/s.

## Author Contributions

EC, as the sole author, collected and analyzed all the data, and was the sole responsible for the conceptualization and writing-up of this manuscript.

## Conflict of Interest

The author declares that the research was conducted in the absence of any commercial or financial relationships that could be construed as a potential conflict of interest.

## Footnotes

^1^Although in a first phase of the pandemic, Portugal and Greece seemed to be effective in managing the health crisis, in subsequent moments of the sanitary crisis, the situation has changed, at least for Portugal, that was not anymore portrayed as a successful case.
