# Supplementary material for: War Metaphors in Political Communication on Covid-19
Source: Front Sociol. 2021 Jan 25;5:583680. doi: 10.3389/fsoc.2020.583680 (PMC8022596; doi:10.3389/fsoc.2020.583680)
Supplement: Supplementary file 1 [file Data_Sheet_1.PDF]

Supplementary Material (Speeches)

Publicly available datasets were analyzed in this study. Below are the links for the analyzed speeches:

Marcelo Rebelo de Sousa (President of Portugal)
18 March 2020

https://www.publico.pt/2020/03/18/politica/noticia/discurso-marcelo-integra-1908422

Emmanuel Macron (President of France)
16 March 2020

https://www.lemonde.fr/politique/article/2020/03/16/nous-sommes-en-guerre-retrouvez-le-discours-de-macron-pour-lutter-contre-le-coronavirus_6033314_823448.html

Declaración institucional/ Pedro Sánchez (Prime-Minister of Spain)
21 March 2020

https://www.youtube.com/watch?v=J7u-b-EoADY

Boris Johnson (Prime-Minister of the UK)
23 March 2020

https://www.gov.uk/government/speeches/pm-address-to-the-nation-on-coronavirus-23-march-2020

Speech by President von der Leyen at the European Parliament Plenary on the European coordinated response to the COVID-19 outbreak
Brussels, 26 March 2020

https://ec.europa.eu/commission/presscorner/detail/en/speech_20_532

António Guterres, the ninth Secretary-General of the United Nations
23 March 2020
Opening remarks of the Secretary-General's appeal for global ceasefire

https://www.un.org/sg/en/content/sg/speeches/2020-03-23/secretary-general-appeal-for-global-ceasefire

António Guterres, the ninth Secretary-General of the United Nations
26 March 2020: Remarks at G-20 Virtual Summit on the COVID-19 Pandemic

https://www.un.org/sg/en/content/sg/speeches/2020-03-26/remarks-g-20-virtual-summit-covid-19-pandemic

Donald Trump (President of the US)

Remarks by president Trump at the coronavirus task force in press briefing.
18 March 2020
https://www.whitehouse.gov/briefings-statements/remarks-president-trump-vice-president-pence-members-coronavirus-task-force-press-briefing-5/

Speech at Virus Task Force Hold Briefing
19 March 2020
https://www.whitehouse.gov/briefings-statements/remarks-president-trump-vice-president-pence-members-coronavirus-task-force-press-briefing-6/

https://www.whitehouse.gov/briefings-statements/
